# Alcohol abuse among women: a review with a gender perspective

**DOI:** 10.1192/j.eurpsy.2022.2122

**Published:** 2022-09-01

**Authors:** J. Gonçalves Cerejeira, I. Santos Carrasco, C. Vallecillo Adame, C. De Andrés Lobo, T. Jiménez Aparicio, M. Queipo De Llano De La Viuda, A. Gonzaga Ramírez, G. Guerra Valera

**Affiliations:** 1 Hospital Clínico Universitario de Valladolid, Psychiatry, Valladolid, Spain; 2 Clinical Hospital of Valladolid, Psychiatry, Valladolid, Spain; 3 Hospital Clínico Universitario, Psiquiatría, Valladolid, Spain; 4 Hospital Clínico Universitario de Valladolid, Psiquiatría, VALLADOLID, Spain

**Keywords:** alcohol abuse, women

## Abstract

**Introduction:**

The harmful use of alcohol is an important risk factor for the health of the population around the world. The incidence of alcohol dependence in women is increasing and both its consumption pattern and its consequences have unique characteristics.

**Objectives:**

To present a literature review focused on alcohol use disorder with a gender perspective.

**Methods:**

Literature review.

**Results:**

- Women use to start using alcohol sooner than men and this seems to be a risk factor to become addicted. - Due to physiological and psychological factors women experience more negative health effects from excessive alcohol use than men and it occurs at lower levels of use. - Psychiatric comorbidity associated with alcohol abuse such as anxiety and depression is more common in women and this in turn worsens the alcohol use disorder. - Alcohol consumption increases the vulnerability of women on several levels, including an increased risk of physical abuse.

**Conclusions:**

Alcohol abuse among women deserves special attention and a specific intervention focused on the gender perspective.

**Disclosure:**

No significant relationships.

